# A nanodroplet cell processing platform facilitating drug synergy evaluations for anti-cancer treatments

**DOI:** 10.1038/s41598-019-46502-3

**Published:** 2019-07-12

**Authors:** Ching-Te Kuo, Jong-Yueh Wang, Siang-Rong Lu, Yu-Sheng Lai, Hsiu-Hao Chang, Jer-Tsong Hsieh, Andrew M. Wo, Benjamin P. C. Chen, Jen-Her Lu, Hsinyu Lee

**Affiliations:** 1 Department of Electrical Engineering, Graduate Institute of Electronics Engineering, National Taiwan University, Taipei, Taiwan; 2 Department of Life Science, National Taiwan University, Taipei, Taiwan; 3 Department of Pediatrics, National Taiwan University Hospital and National Taiwan University College of Medicine, Taipei, Taiwan; 4 Department of Urology, University of Texas Southwestern Medical Center, Dallas, TX USA; 5 Institute of Applied Mechanics, National Taiwan University, Taipei, Taiwan; 6 Division of Molecular Radiation Biology, Department of Radiation Oncology, University of Texas Southwestern Medical Center, Dallas, TX USA; 7 Department of Pediatrics, Taipei Veterans General Hospital, Taipei, Taiwan; 8 School of Medicine, National Yang-Ming University, Taipei, Taiwan

**Keywords:** High-throughput screening, Biomedical materials, Chemotherapy

## Abstract

Therapeutic drug synergism intervened in cancer treatments has been demonstrated to be more effective than using a single effector. However, it remains inherently challenging, with a limited cell count from tumor samples, to achieve potent personalized drug cocktails. To address the issue above, we herein present a nanodroplet cell processing platform. The platform incorporates an automatic nanodroplet dispenser with cell array *ParaStamp* chips, which were fabricated by a new wax stamping approach derived from laser direct writing. Such approach enables not only the on-demand de-wetting with hydrophobic wax films on substrates but also the mask-less fabrication of non-planar microstructures (i.e. no photolithography process). The *ParaStamp* chip was pre-occupied with anti-cancer drugs and their associate mixtures, enabling for the spatially addressable screening of optimal drug combinations simultaneously. Each droplet with a critical volume of 200 nl containing with 100 cells was utilized. Results revealed that the optimal combination reduces approximate 28-folds of conducted doses compared with single drugs. Tumor inhibition with the optimally selected drug combination was further confirmed by using PC-3 tumor-bearing mouse models. Together, the nanodroplet cell processing platform could therefore offer new opportunities to power the personalized cancer medicine at early-stage drug screening and discovery.

## Introduction

Synergistic combination of two or more drugs has been a major avenue targeting cancers^1,2^. This regimen not only improves the therapeutic efficacy by triggering synthetic lethality in target cells but also minimizes the side effects by reducing doses of each drug^3,4^. Therefore, the identification of optimal combination of various possible concentrations from a set of drugs presents a substantial challenge.

Several approaches to optimize the selection regime have been demonstrated, in terms of large scale simulations^5,6^ and stochastic search algorithms^7,8^. The power of feedback system control (FSC) methodology can facilitate the screenings down to 10–20 iterative tests out of million possible combinations; however, challenges still remain^9^. For example, the time for cell preparation adopted among the total independent iterations would last for weeks. In addition, the usage of conventional multi-well plate assays would counter the feasibility for personalized medicine, which is inherently subject to a limited cell count from tumor samples^10^. Although the multilayered culture technique has been demonstrated to better predict the *in vivo* efficacy targeting tumor microenvironments, the time, required cell amount and complexity of experimental setup are not addressed as well^11^. As such, a new technique needs to be addressed against the shortcomings described above.

In the past decade, cell-based microarray platforms have been demonstrated to address the issues of critical drug volume, cell-source limitation, or high-throughput and high-content screening^12–17^. Notably, a recently developed approach involved cell microarray with cancer stem cells (CSCs) to potentially address the tumor heterogeneity *in vivo*^18^. Likewise, other researches revealed that cells cultured in microscales could contribute distinct drug responses, attributing to the higher cell to volume ratio compared with macroscale cultures^19,20^. Although the translation from *in vitro* to *in vivo* optimal drug combinations has been successfully performed with standard multi-well plates, relatively little effort has been directed toward using cell arrays as biosensor tools for pre-clinical *in vivo* assays^8,11,21^.

To address the technology gap described above, we herein present the nanodroplet cell processing platform for high-throughput screenings of optimal drug combinations. The platform incorporates a programmable nanodroplet dispenser with *ParaStamp* cell array chips. The chips were fabricated following a new mask-less approach developed to improve the hydrophobic wax stamping with well arrays with a uniform size (i.e. without photolithography process). We demonstrated that approximate 500-fold miniaturization does not impact the *in vivo* outcome (81 test spots per 22 × 22mm^2^; 100 cells in 200 nl per spot). Taken together, these findings highlight our newly developed nanodroplet cell processing platform could become a cost-effective, purpose-tailored and high-throughput toolkit for improving pre-clinical drug screening efficacy.

## Results and Discussion

### Characteristics of polydimethylsiloxane (PDMS) *ParaStamp*

**Figure 1:**
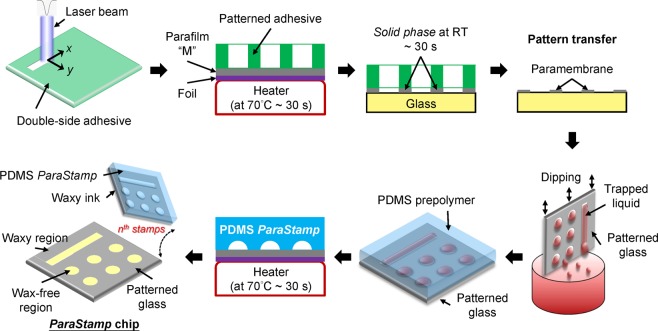
PDMS *ParaStamp* and *ParaStamp* chip. Illustration shows the fabrication process of PDMS *ParaStamp* and *ParaStamp* chip.

**Figure 2:**
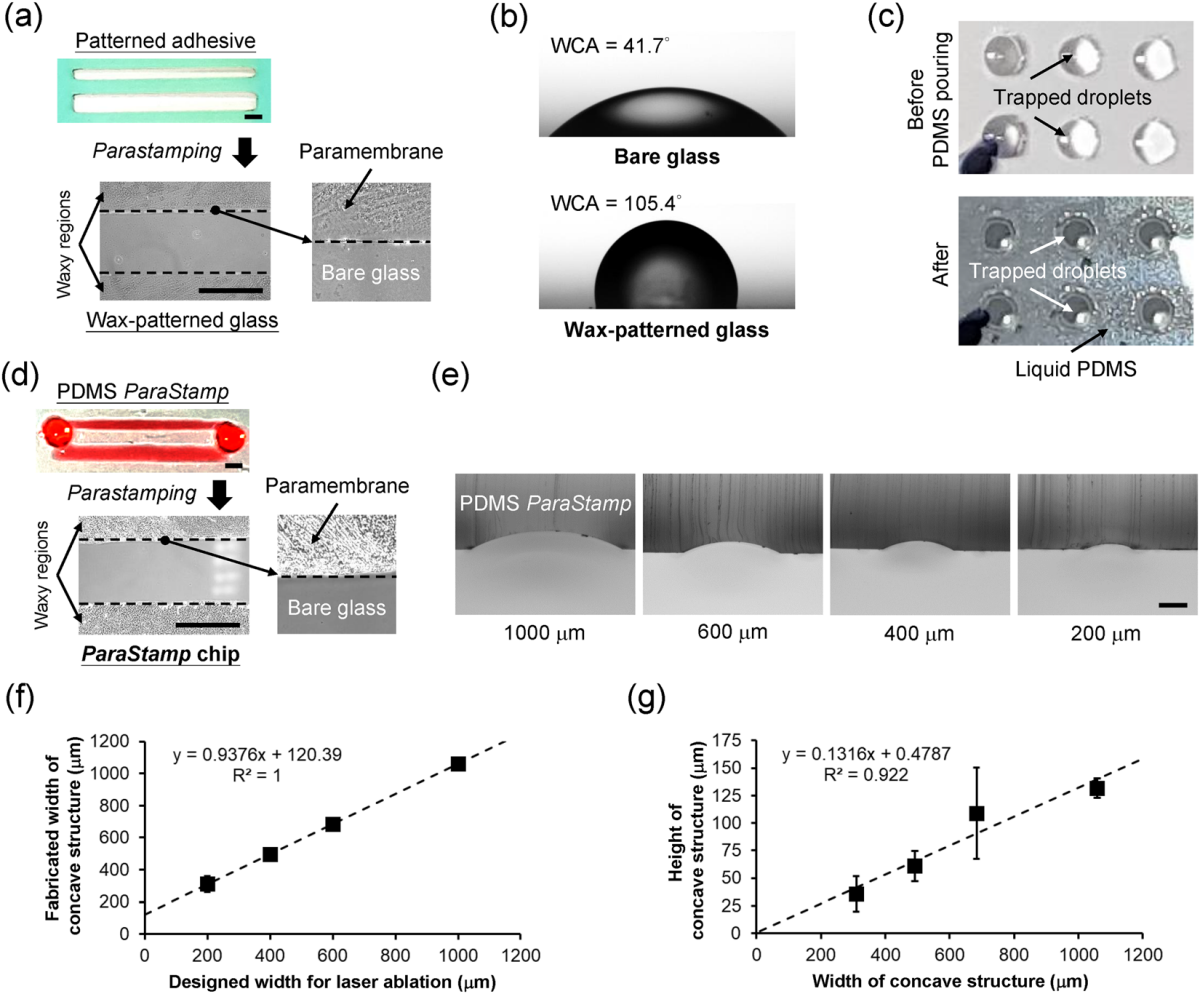
Characteristics of PDMS *ParaStamp* and *ParaStamp* chip. (**a**) shows the patterned adhesive by CO2 laser ablation and its representative micrograph of stamped pattern with waxy paramembrane, respectively. Scale bar, 1 mm. (**b**) Measurements of water-contact angle (WCA) for bare and wax-stamped glasses, respectively. The volume of each droplet is 1 μl. (**c**) Photographs showing the trapped droplets on *ParaStamp* chips before and after liquid PDMS poruing, respectively. (**d**) shows the fabricated PDMS *ParaStamp* and its representative micrograph of stamped pattern with waxy paramembrane, respectively. The pattern of *ParaStamp* is highlighted by red inks. Scale bar, 1 mm. (**e**) Micrographs show the concave microstructures of fabricated *ParaStamp* with different dimensions. Scale bar, 200 μm. (**f**) Relationship between the designed width for laser ablation and the concave width of fabricated *ParaStamp*. (**g**) The fabricated dimensions (width and height) in *ParaStamp* behave in a linear relationship (mean ± SD, n = 3 except n = 2 for 200 μm of designed width).

We incorporated a mask-less approach with a laser direct-writing availability in four steps to fabricate the PDMS stamp (Fig. 1): programmable engraving on adhesive tapes with a CO_2_ laser; stamping a hydrophobic paraffin wax with corresponding patterns from the engraved tapes on glass substrates [a representative image shown in Fig. 2(a) and the water contact angles are 105.4° and 41.7° for patterned substrate and bare glass, respectively, shown in Fig. 2(b)]; placing the patterned glasses in a water bath to enable the sessile droplets to spontaneously reflect the underlying patterns [a representative image shown in Fig. 2(c)]; and casting the concave microstructures by PDMS prepolymers to present the *ParaStamp* with repeatable stamping ability [a representative image shown in Fig. 2(d)]. We demonstrated that the PDMS *ParaStamp* could be achieved with various characteristic sizes [Fig. 2(e)], in which the sizes behave in a linear relationship depending on the designed parameters for laser micromachining [Fig. 2(f)]. In addition, the aspect ratio of the PDMS *ParaStamp* was obtained with an approximate 0.13 of height to width [Fig. 2(g)].

CO_2_ laser micromachining has been widely adopted in biomedical applications, owing to its rapid and mask-less prototyping potentials^22–24^. In this paper, we first and successfully demonstrate that such the micromachining can be applied to facilitate the *ParaStamp* technique^25^. It could achieve the critical size of PDMS *ParaStamp* fabricated down to 120 μm [Fig. 2(f)], which is parallel to the typical trench width with 140 μm engraved in poly(methyl methacrylate) (PMMA)^22^. Most importantly, the PDMS *ParaStamp* could be re-operated up to 50 times without destroying the patterns (data not shown here). These highlight the power of using PDMS *ParaStamp* for a wide range of applications, e.g. cell patterning and rewritable droplet storage as well as cost-effective drug synergy screening^25^.

### High-throughput drug synergy screening *via* the nanodroplet cell processing platform

**Figure 3:**
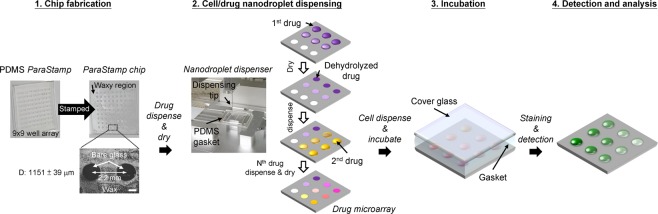
Procedure of multiple drug dispensing, cell loading and evaluation of drug toxicity by the nanodroplet cell processing platform. The left top panel shows the fabricated *ParaStamp* and the corresponding 9 × 9 *ParaStamp* chip on a 22 × 22 mm^2^ cover glass, respectively. The left bottom panel shows the representative micrograph of two circular wax-patterned wells. The diameter (D) of the wells are 1151 ± 39 μm (coefficient of variation, CV = 3.4%, n = 32). The volume of each droplet dispensed is 200 nl. Scale bar, 500 μm.

**Figure 4:**
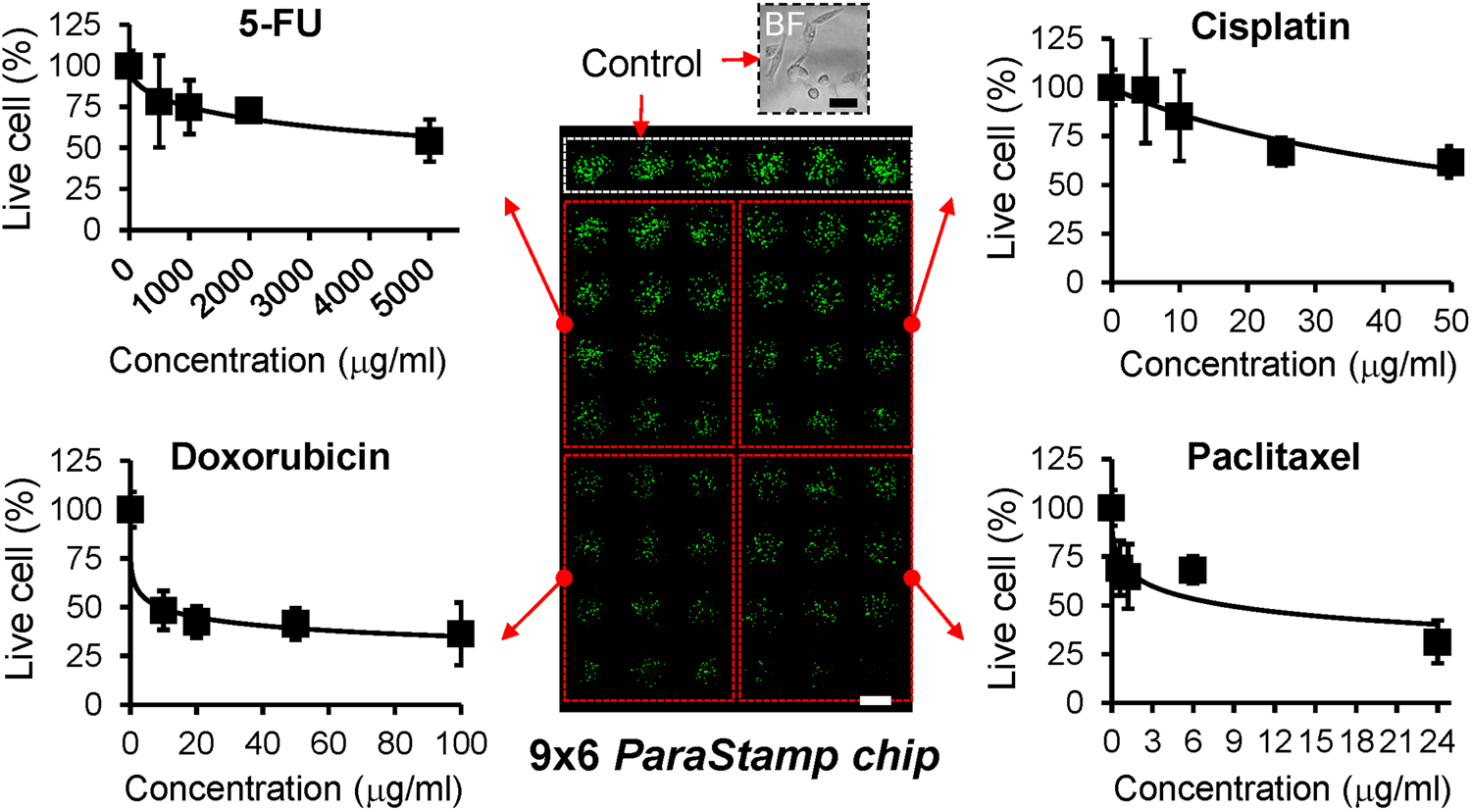
High-throughput drug screening *via* the nanodroplet cell processing platform. Toxicity profiles of 24 h 5-FU, cisplatin, doxorubicin and paclitaxel treatments on PC-3 cells, derived from a 9 × 6 *ParaStamp* chip. Live cells were detected by Calcein AM labeling (green color). Each data represents the mean ± SD from 2 ~ 3 independent experiments (n = 6 ~ 9). Scale bar, 1 mm; insert bar in bright field (BF), 50 μm.

It has been demonstrated with a potential to improve the therapeutic relevant selectivity by synergistic drug combinations. Our developed nanodroplet platform also facilitates the drug synergy screening from the proof-of-conceptual experiment, as illustrated in Figs 3 and 4 (see also Supplementary Fig. S1). We applied the PDMS *ParaStamp* to fabricate 9 × 9 well arrays on a glass substrate (i.e. *ParaStamp* chip), in which the average diameter of patterned wells is 1151 μm with a coefficient of variation (CV) to be 3.4%. The *ParaStamp* chip contains arrayed dehydrolyzed drugs, in which each drug droplet (200 nl) is first dispensed with a single drug or a mixture of drugs with different concentrations and then dried. A PDMS-glass gasket was applied to prevent the evaporation of nanodroplets during experiments (Supplementary Fig. S2). To direct the advantage of using such chip, we first treated prostate PC-3 cancer cells with different concentrations of four commonly used chemotherapeutic drugs – cisplatin, paclitaxel, 5-FU and doxorubicin for a 1-day single drug treatment (Fig. 4). We observed that the IC_50_ values derived from the chip were 62.9 ± 5.0, 8.0 ± 0.1, 8324.2 ± 2475.2 and 13.4 ± 2.2 μg/ml for cisplatin, paclitaxel, 5-FU and doxorubicin, respectively.

**Figure 5:**
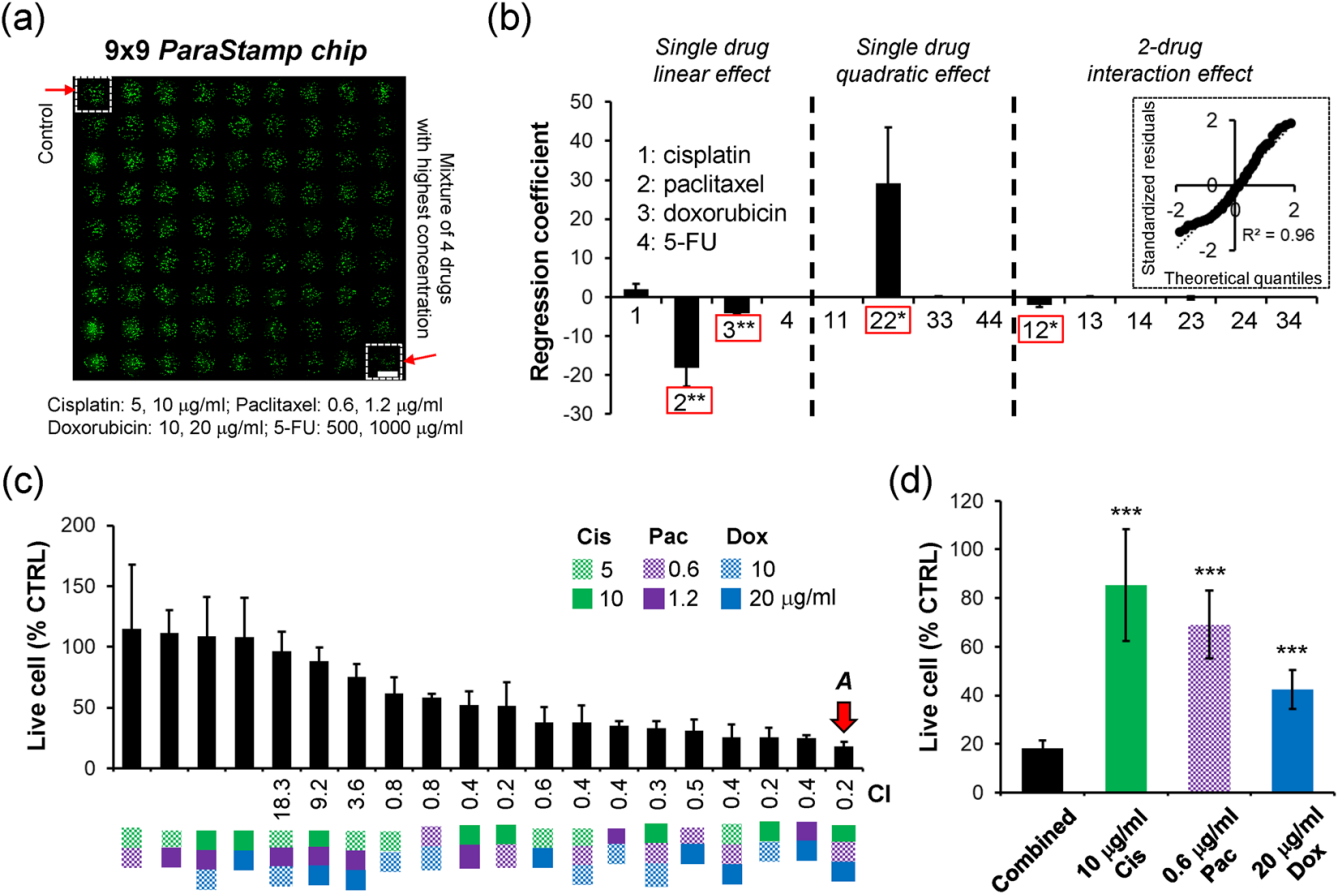
Selection of optimal chemotherapeutic drug combinations using the nanodroplet cell processing platform. (**a**) Fluorescent detection of live PC-3 cells by Calcein AM labeling (green color) under the treatments with cisplatin (5 and 10 μg/ml), paclitaxel (0.6 and 1.2 μg/ml), doxorubicin (10 and 20 μg/ml), 5-FU (500 and 1000 μg/ml), and their combinations after 24 h. Details can be referred in Supplementary Table 1. Scale bar, 1 mm. (**b**) Regression coefficients evaluated from the stepwise linear regression model. (**c**) Cell viabilities of PC-3 cells under the combinational treatments of cisplatin (Cis), paclitaxel (Pac) and doxorubicin (Dox) with different concentrations. The selected drug optimization is highlighted by the red arrow. Each data represents the mean ± SD from 3 independent experiments (*p < 0.05 and **p < 0.01). (**d**) Comparison of cell viabilities obtained from the single-drug treatment and the optimal combination (mean ± SD from 2~3 independent experiments; ***p < 0.001).

For demonstration the drug synergy screening, we treated PC-3 cells with three different concentrations of the four anti-cancer drugs described above and their associated mixtures [Fig. 5(a)]. It therefore yielded totally 81 drug combinations, including control, 5 and 10 μg/ml of cisplatin, 0.6 and 1.2 μg/ml of paclitaxel, 500 and 1000 μg/ml of 5-FU, 10 and 20 μg/ml of doxorubicin, and the rest 72 associate mixtures. Based on the first screening with regression coefficient, we further determined cisplatin, paclitaxel, and doxorubicin as the potent effectors to inhibit PC-3 cell proliferation [Fig. 5(b)]. The optimal drug combination was selected to be 10 μg/ml cisplatin, 0.6 μg/ml paclitaxel and 20 μg/ml doxorubicin, which is depending on the second screening with combinational synergy from the 3-drug cocktails [Fig. 5(c)]. This optimum was demonstrated to be more efficient than single drug treatment [Fig. 5(d)]. Moreover, it could reduce the single drug doses of cisplatin, paclitaxel, and doxorubicin down to 45.4, 34.4 and 5.3 folds, respectively, under the 82% optimal cell inhibition. It therefore contributed an average 28.4-fold reduction among the three drugs (combination *A* in Supplementary Table S1).

In this paper, we conducted a drug pre-coating approach to facilitate the drug screening with the chip, thus excluding the issue of droplet evaporation during the sequenced dispensing with drugs. Where it may cause the distinction while the aqueous drugs turn into dried solids, possibly lacking their pharmaceutical activity. We therefore performed an experiment to compare our approach used and the conventional adding-with-drug in 96-well plates. Results revealed that no significant difference is detected between the two approaches (Supplementary Fig. S3). It therefore highlights that our proposed approach could be applied for a potential live-cell biosensor that is pre-occupied with drugs.

### Partially successful translation from *in vitro* drug combination to *in vivo* drug administration

To demonstrate the feasibility of using the nanodroplet platform to predict the *in vivo* efficacy, we first employed PC-3 tumor-bearing mouse models to compare the three different drug combinations screened from *in vitro* assays (combination *A* in Supplementary Table S1). Based on an empirical correlation, one could convert the drug doses to be administered between animals and humans. However, there is relatively less proper formula to systematically translate the *in vitro* drug doses to *in vivo* sets^8,11,21,26^. We therefore developed a translated algorithm, as exhibited in Eqs (2) and (3), to be applied into the *in vivo* models (details can be referred in Methods). The translated doses were listed in Supplementary Table S1.

**Figure 6:**
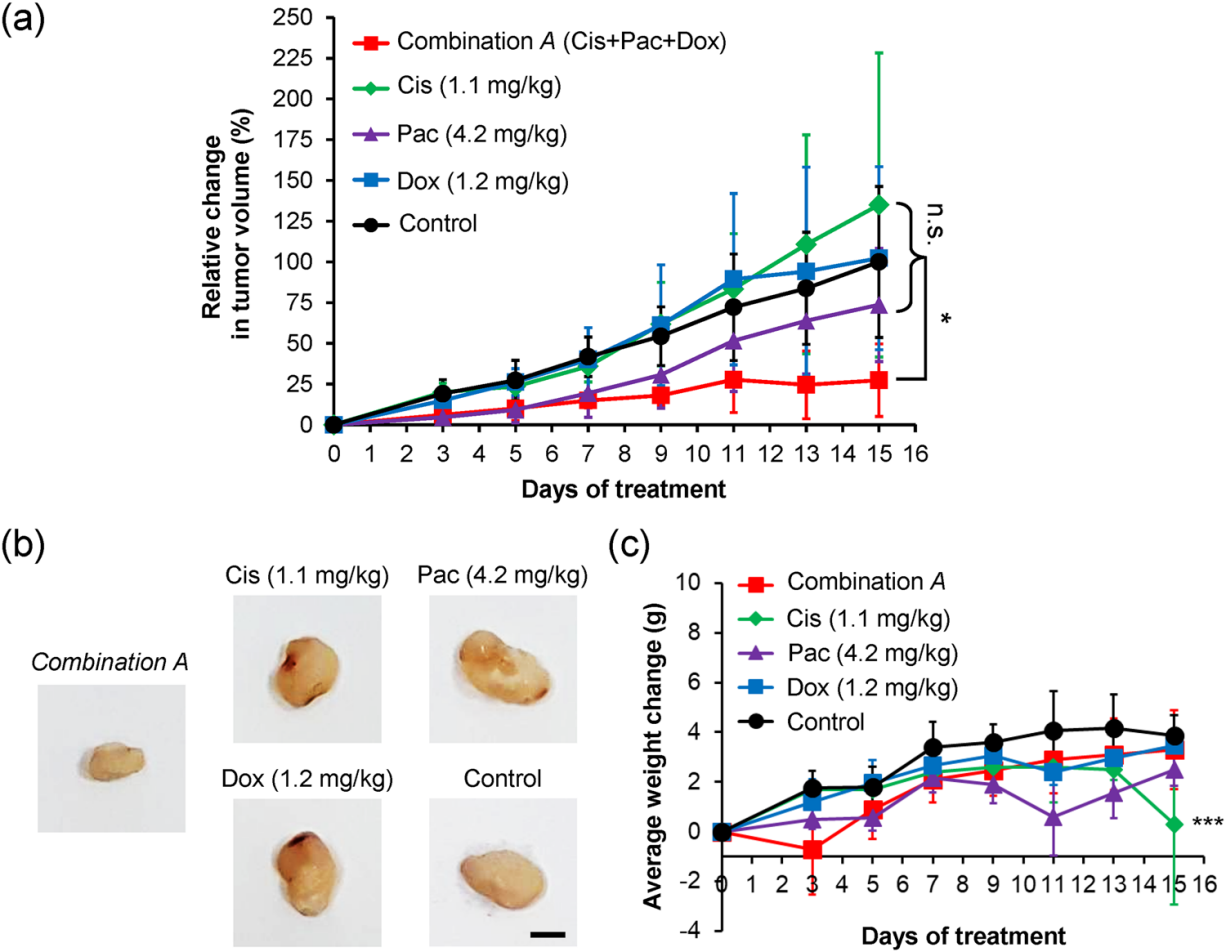
Inhibition of PC-3 tumor growth in athymic nu/nu (nude) mice by the optimal drug combination. (**a**) Tumor inhibitions by the selected drug combination *A* (derived from the nanodroplet platform). The combination *A* includes 1.1 mg/kg cisplatin, 4.2 mg/kg paclitaxel, and 1.2 mg/kg doxorubicin. The control sets were administered with PBS. The change in tumor volume was evaluated as a volume change against the initial volume at day 0. Each data represents the mean ± SD as a percentage of the final volume change of controls at day 15 (n = 4 except n = 2 for Cis; *p < 0.05 (with a power = 0.65)). Two mice were dead during the Cis-treated experiments. (**b**) Representative photographs of tumors treated with combination *A*, single drug doses and PBS as the control at the last experiment day (day 15). Scale bar, 5 mm. (**c**) Average body weight change during the drug administrations. The weight change was against the initial weight at day 0 (mean ± SD, n = 4 except n = 2 for Cis; *p < 0.05, **p < 0.01, and ***p < 0.001).

Results of the mouse model assays showed that the screened combination *A* presents a tumor inhibition (p < 0.05) compared with the control, whereas no difference is observed among the single drugs administered [Fig. 6(a,b)]. For the requirement of power = 0.8, the total sample size needed was calculated to be 25 mice, which is larger than 18 mice as power = 0.65 from our experimental data at Day 15 (see also the section of Power analysis in the Supplementary Information). In addition, there was no significant weight change of mice among the individual drug treatments except for the cisplatin treatment at Day15 [Fig. 6(c)].

We further compared the *in vivo* prediction of drug responses derived from our platform and the standard 96-well plate assay (Supplementary Fig. S4). Both of them presented the same *in vitro* drug combinations, similar responses in cell viability (18.2% from platform and 20.0% from plate) and similar synergistic CI values (0.2 from platform and 0.5 from plate), as listed in Supplementary Table S1. The drug doses translated from the platform (combination *A*) to mice were determined to be 1.1 mg/kg cisplatin, 4.2 mg/kg paclitaxel and 1.2 mg/kg doxorubicin, whereas the plate (combination *B*) was obtained with 4.7 mg/kg cisplatin, 17.9 mg/kg paclitaxel and 5.3 mg/kg doxorubicin. Although the three *in vivo* drug doses in combination *B* were particularly 3-folds larger than that in *A*, the efficacy of tumor inhibition from *A* was significantly higher than *B* (Supplementary Fig. S4). Indeed, the single drug treatment with a higher concentration would lead a slower tumor growth (Supplementary Fig. S5). In other words, it indicates that the nanodroplet platform could predict the *in vivo* drug efficacy more efficiently than the 96-well plate assay.

Previous studies have revealed that the synergistic drug combinations, derived from well-plate assays, can benefit the therapeutic outcomes^2,8,9^. In this paper, we furthermore demonstrate that our nanodroplet cell processing platform could not only streamline the screening of potent drug combinations within one day, but also properly predict the *in vivo* efficacy. In addition, we reveal that approximate 500-fold miniaturization (200 nl of volume in platform *versus* 100 μl in 96-well plate per test) does not impact the *in vivo* outcome (Fig. 6 and Supplementary Fig. S4). Its critical volume needed per test (200 nl) is significantly smaller than 1.5 μl of recommended working volume for Corning 1536 well plates. Moreover, the optimal drug combination derived from our platform could reduce approximately 28-folds of conducted doses compared with single drugs used. Our findings herein imply that our developed platform could further ignite new applications for drug screening on rare cells, e.g. CSCs or circulating tumor cells (CTCs)^27^.

## Conclusions

In conclusion, we have presented the nanodroplet cell processing platform – automatic nanodroplet dispensing and *ParaStamp* cell array chips) – in which the chips were fabricated by a new methodology by using laser direct writing-derived wax stamping. The new approach has been successfully demonstrated to facilitate not only the on-demand surface patterning with hydrophobic wax films but also the mask-less fabrication of concave microstructures. In addition, we successfully demonstrate that the nanodroplet platform can be applied for the high-throughput drug synergy screening *in vitro* and *in vivo*. Beyond the above concerns, we anticipate that the new wax patterning technique and its resulted bio-assay cell array chip will direct a facile and effective avenue to achieve the high-throughput requirement for future biomedical applications.

## Methods

### Preparation of PDMS *ParaStamp* and *ParaStamp* chip

The PDMS *ParaStamp* was prepared as the schematic shown in Fig. 1. First, a thermally conductive adhesive tape (8805, 3M^TM^) was patterned by design and ablated by a CO_2_ laser engraver (Mercury II, LaserPro Inc. Grand Prairie, TX). Second, a layer of hydrophobic/de-wetting paraffin wax was patterned on a plain glass slide or a cover glass (with a 22 × 22 mm^2^ of total area), following the below approach. A commercial Parafilm “M” was tailored and sandwiched by a foil and the patterned adhesive prior to heating at 70 °C for 30 s. The patterned adhesive was peeled off and stamped on a glass substrate followed by cooling for another 30 seconds at room temperature. After peeling off the adhesive stamp, the wax patterns transferred was achieved. Third, the aqueous mold with patterns was simultaneously performed by dipping and then slowly pulled with the wax-patterned glass substrate from a water bath. Afterwards, the corresponding microstructures with concave features were cast by PDMS prepolymers at 70 °C for 1 h, leading the formation of a PDMS *ParaStamp* and its resulted *ParaStamp* chips. The weight ratio of PDMS base to curing agent was 10:1. The patterned adhesive can only be utilized to transfer the corresponding wax patterns for one time, due to the thermal deformation of itself. Instead, the PDMS *ParaStamp* can be repeatedly used for various substrates on demand because of its thermal resistance.

### Cell line

Human prostate cancer cell line PC-3 (CRL-1435, ATCC, Manassas, VA) was maintained in RPMI-1640 medium (SH30027.02, HyClone), supplemented with 10% fetal bovine serum (FBS; 10437028, Gibco) and 1% penicillin/streptomycin (P/S; GT-SPS100, GeneTeks Bioscience), and cultured in a humidified 5% CO_2_ incubator at 37 °C.

### High-throughput drug synergy screening

To demonstrate the feasibility of drug synergy screening *via* the nanodroplet platform, we fabricated a 9 × 9 *ParaStamp* chip following the procedure as shown in Fig. 1. The array chip contained total 81 paraffin wax-made wells patterned on a 22 × 22 mm^2^ cover glass, in which the diameter of the wells (bare glass surfaces for cell culturing) is 1151 μm and the center-to-center distance is 2.2 mm (Fig. 3). The chip was sterilized by UV for 30 min prior to use.

For the high-throughput drug synergy screening, we followed the procedure shown in Fig. 3. Prostate PC-3 cells were conducted under the single or synergistic effects of four chemotherapeutic drugs – cisplatin (Fresenius kabi, Solan, Himachal Pradesh, India), paclitaxel (Phyxol; Sinphar Pharmaceutical, Taiwan), 5-FU (Haupt Pharma, Wolfratshausen, Germany), and doxorubicin (Adriamycin, Pfizer, New York, NY). First, cisplatin droplets with different concentrations were dispensed onto the corresponding wells by using a programmable nanodroplet dispensing machine (Versa 10 spotter, Aurora Instruments Ltd. Vancouver, CA). Drugs were diluted in deionized (DI) water and the volume of each droplet was 200 nl. The array chip was then incubated at room temperature for 10 minutes, allowing the droplets to evaporate and therefore to convert into drug powders accordingly. Subsequently, paclitaxel, 5-FU, and doxorubicin drugs were dispensed following the approach described above, respectively. Afterwards, cell droplets with a volume of 200 nl (100 cells per droplet) were dispensed onto the array chip, followed by assembling with a PDMS-glass gasket cover, and then incubated for one day. After the 1-day drug treatment, cell viability was determined by staining live cells with Calcein AM (4 μM; Invitrogen, Carlsbad, CA). The fluorescence intensities of live cells were captured by a charge-coupled device (CCD) camera mounted on an upright microscope, and analyzed by a Fiji imaging macro software. The live cell percentage, according to the detected fluorescence intensity, was normalized against the untreated cells. The IC_50_ values were fitted and evaluated by a four-parameter logistic equation presented^28^.

For drug screening performed in 96-well plates, media containing PC-3 cells with a density of 3000 cells per well were first conducted. The concentrated drug solutions were then added into the corresponding wells, leading the same drug concentrations compared with that used in nanodroplet platform and a final volume with 100 μl per well. The cell viability was evaluated from a plate reader (TCX-LS07, NTU) based on MTT (3-[4,5-dimethylthiazol-2-y1]-2,5-diphenyl tetrazolium bromide) assay.

### Linear regression model

The stepwise model was based on a linear regression equation:

1 $$y={b}_{0}+\sum _{i=1}^{k}{b}_{i}{x}_{i}+\sum _{i=1}^{k}{b}_{ii}{{x}_{i}}^{2}+\sum _{i=1}^{k}\sum _{j=1}^{k}{b}_{ij}{x}_{i}{x}_{j}+c$$

where *y* is the cell viability. *b*_0_, *b*_*i*_, *b*_*ii*_ and *b*_*ij*_ are the intercept, single drug linear interaction, quadratic and bilinear 2-drug interaction terms, respectively. *x* represents the drug dose. *c* is the error term with a mean equaling to zero^2^.

### Combination index (CI)

To determine the effect of drug combination synergy, the CI values evaluated from the CompuSYN software were adopted to represent the synergistic interaction (CI < 0.8), the additive effect (0.8 ≤ CI ≤ 1) or the antagonism (CI > 1).

### Translating of selected *in vitro* drug combinations to *in vivo* drug administrations

The translation from *in vitro* to *in vivo* drug doses was based on the formula we utilized in this work:

2 $${D}_{x,invivo}=(\frac{MT{D}_{x}}{DR{I}_{ave}})\times n$$

where *x* is the specific drug selected from the *in vitro* combinations, *D*_*x,in vivo*_ is the drug dose evaluated for the *in vivo* drug *x* administration, *MTD*_*x*_ is the maximum tolerated dose of drug *x* used for the *in vivo* assay, and *n* is the total number of drug administrations during the period of the assay. *DRI*_*ave*_ is the average of dose reduction indexes (DRIs), which is calculated from

3 $$DR{I}_{ave}=\frac{\sum _{x=1}^{m}DR{I}_{x}}{n}$$

where *DRI*_*x*_ is the DRI value of drug *x*, which is determined from the CompuSYN software based on the cell viability data (Fig. 4), and *m* is the number of total drugs *x* used.

### *In vivo* subcutaneous xenograft model assay

Male athymic nu/nu (nude) mice aged 4–6 weeks were obtained from Lasco Biotech Industry (weight 20–25 g). Each mouse was injected subcutaneously with a total number of 3 × 10^6^ Matrigel-encapsulated PC-3 cells. Tangible tumors were visible within 5–7 days, at which time the drug administration by intraperitoneal (IP) injection was initialized. Each drug combination or single drug was administered by multiple injections of the translated *in vivo* dose at days 0, 3, 5, 7, 9, 11, and 13 to mice (i.e. totally seven time points), at which time the tumors were scored. Each experimental group was applied with four mice. The tumor volume *V* was evaluated by the equation V = 4 × π × (l/2)^3^/3, in which the mean diameter *l* = (*a* × *b*)^1/2^ (*a* and *b* denote the two orthogonal diameters of each tumor). We compared two different drug combinations *A* and *B* to determine the effective tumor inhibition under the selected drug optimizations (Supplementary Table S1). The drug combination *A* was derived from the nanodroplet platform. In contrast, the combination *B* was from the 96-well plate assay. The average DRIs (*DRI*_*ave*_) were evaluated from the equation (3), which results in (45.4 + 34.4 + 5.3)/3 = 28.4 and (5.1 + 18.6 + 4.0)/3 = 9.2 for the combinations *A* and *B*, respectively. The MTDs of nude mice treated with cisplatin, paclitaxel or doxorubicin were typically at the range of 4.0–6.6 mg/kg^29,30^, 15–36 mg/kg^31,32^ and 2–10 mg/kg^30,31^ per assay, respectively. Based on the Eq. (2), it leaded the *D*_*cisplatin,in vivo*_, *D*_*paclitaxel,in vivo*_ and *D*_doxorubicin*,in vivo*_ from the combination *A* to be 1.0–1.6 mg/kg, 3.7–8.9 mg/kg and 0.5–2.5 mg/kg, respectively. Similarly, the translated doses from the combination *B* were 3.0–5.0 mg/kg (cisplatin), 11.4–27.3 mg/kg (paclitaxel) and 1.5–7.6 mg/kg (doxorubicin). We therefore determined the *in vivo* drug doses based on the selecting margin described above (Supplementary Table S1). Note that, the selecting criterion of the translated dose should not exceed the MTD of each drug. In addition, the drug combinations in *A* and *B* expressed the same doses *in vitro* but not *in vivo*.

### Statistical analysis

To compare data from two groups, Student’s t test was used. For multiple groups comparison, one-way ANOVA test was used. Two-way ANOVA test was adopted to compare data in subcutaneous xenograft model assay. A statistical significance was considered as p < 0.05.

### Compliance with ethic requirements

All institutional and national regulations for the care and use of laboratory mice were followed. All relevant experiments were performed according to the experimental protocols approved by the Animal Care and Use Committee of National Taiwan University, Taiwan.

## Supplementary information

Supplementary information

## Data Availability

Data in this paper will be available after a kind request from the corresponding authors (C.T.K., J.H.L. and H.L.).
