# Supplementary material for: A nanodroplet cell processing platform facilitating drug synergy evaluations for anti-cancer treatments
Source: Sci Rep. 2019 Jul 12;9:10120. doi: 10.1038/s41598-019-46502-3 (PMC6625988; doi:10.1038/s41598-019-46502-3)
Supplement: Supplementary file 1 — Supplementary information [file 41598_2019_46502_MOESM1_ESM.doc]

Supplementary Information for

**A nanodroplet cell processing platform facilitating drug synergy evaluations for anti-cancer treatments**

Ching-Te Kuo^1,2*,**^, Jong-Yueh Wang^2*^, Siang-Rong Lu^2,3^, Yu-Sheng Lai^2^,

Hsiu-Hao Chang^3^, Jer-Tsong Hsieh^4^, Andrew M. Wo^5^,

Benjamin P. C. Chen^6^, Jen-Her Lu^7,8**^ and Hsinyu Lee^1,2**^

*^1^Department of Electrical Engineering, Graduate Institute of Electronics Engineering, National Taiwan University, Taipei, Taiwan*

*^2^Department of Life Science, National Taiwan University, Taipei, Taiwan*

*^3^Department of Pediatrics, National Taiwan University Hospital and National Taiwan University College of Medicine, Taipei, Taiwan*

*^4^Department of Urology, University of Texas Southwestern Medical Center, Dallas, TX, USA*

*^5^Institute of Applied Mechanics, National Taiwan University, Taipei, Taiwan*

*^6^Division of Molecular Radiation Biology, Department of Radiation Oncology,*

*University of Texas Southwestern Medical Center, Dallas, TX, USA*

*^7^Department of Pediatrics, Taipei Veterans General Hospital, Taipei, Taiwan*

*^8^School of Medicine, National Yang-Ming University, Taipei, Taiwan,*

*^*^These authors contributed equally to this work.*

*^**^Dr. Ching-Te Kuo. E-mail: andyjfykuo@gmail.com*

*^**^Prof. Jen-Her Lu. E-mail: jenherlu@gmail.com*

*^**^Prof. Hsinyu Lee. E-mail: hsinyu@ntu.edu.tw*

Supplementary information file includes 1 table and 5 figures.

**Power analysis**

For the examination of power analysis, we adopted a statistical software, the G*Power 3.1.9.4 ([http://www.gpower.hhu.de](http://www.gpower.hhu.de/)), to determinate the power and the corresponding sample size needed. Below figure shows the overall setup for the calculation of power and sample size based on our experimental data from Figure 6(a) at Day 15.


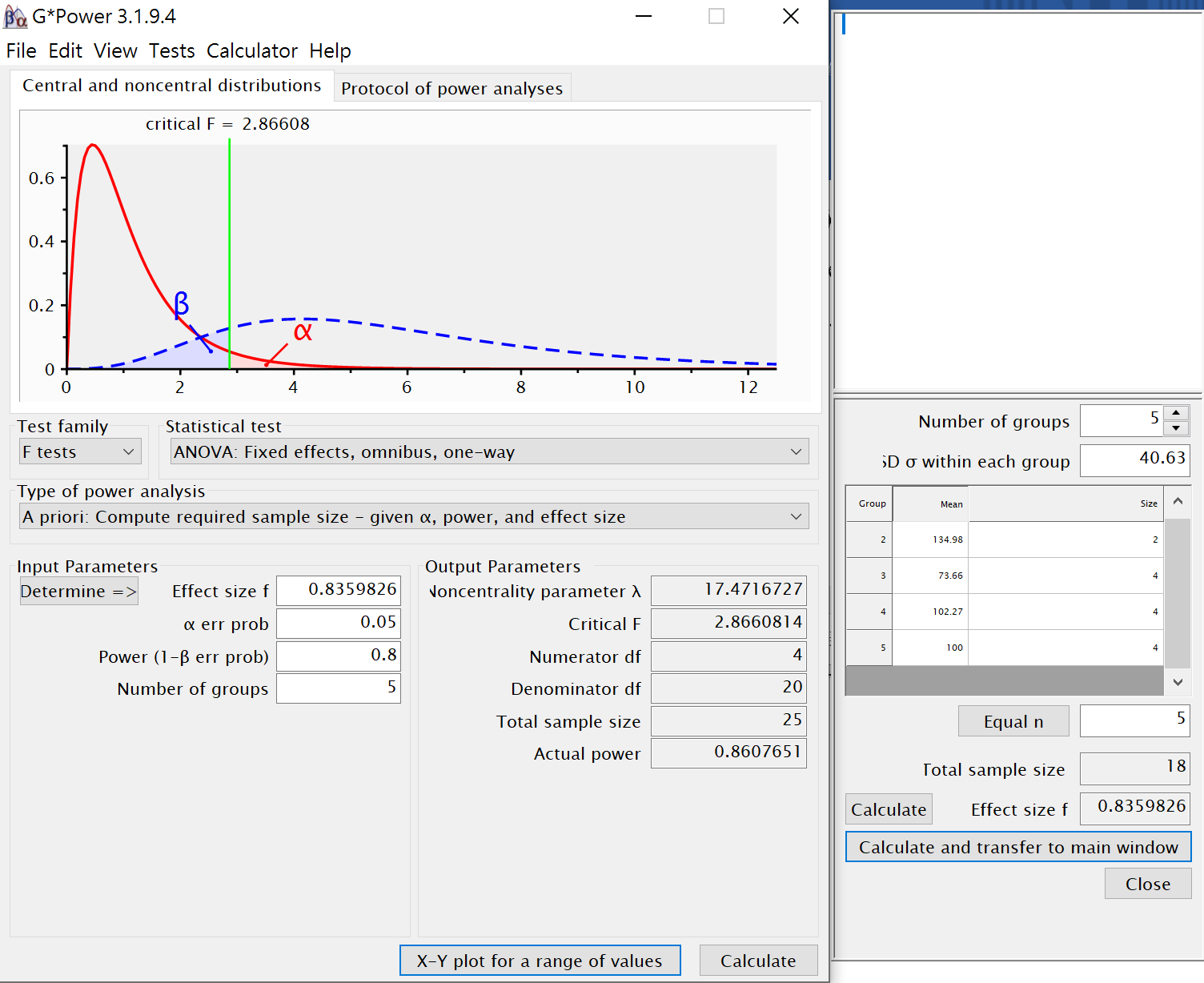


The means of relative tumor volumes treated by combination *A*, cisplatin, paclitaxel, doxorubicin and control were 27.37% (n = 4), 134.98% (n = 2), 73.66% (n = 4), 102.27% (n = 4) and 100% (n = 4), respectively. The relevant standard derivations were 22.25%, 93.31%, 34.80%, 56.13% and 46.31%, respectively. The standard derivation SD, σ, within each group could be evaluated to be 40.63 as following


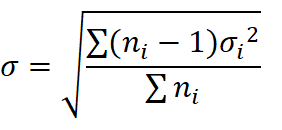

Where *n_i_* is the size of sample group *i* and *s_i_* is the standard derivation of sample *i*. The effective size, f, was then calculated to be 0.8359826. Finally, the relationship between the total sample size needed and the power (1-β) was calculated and present as following


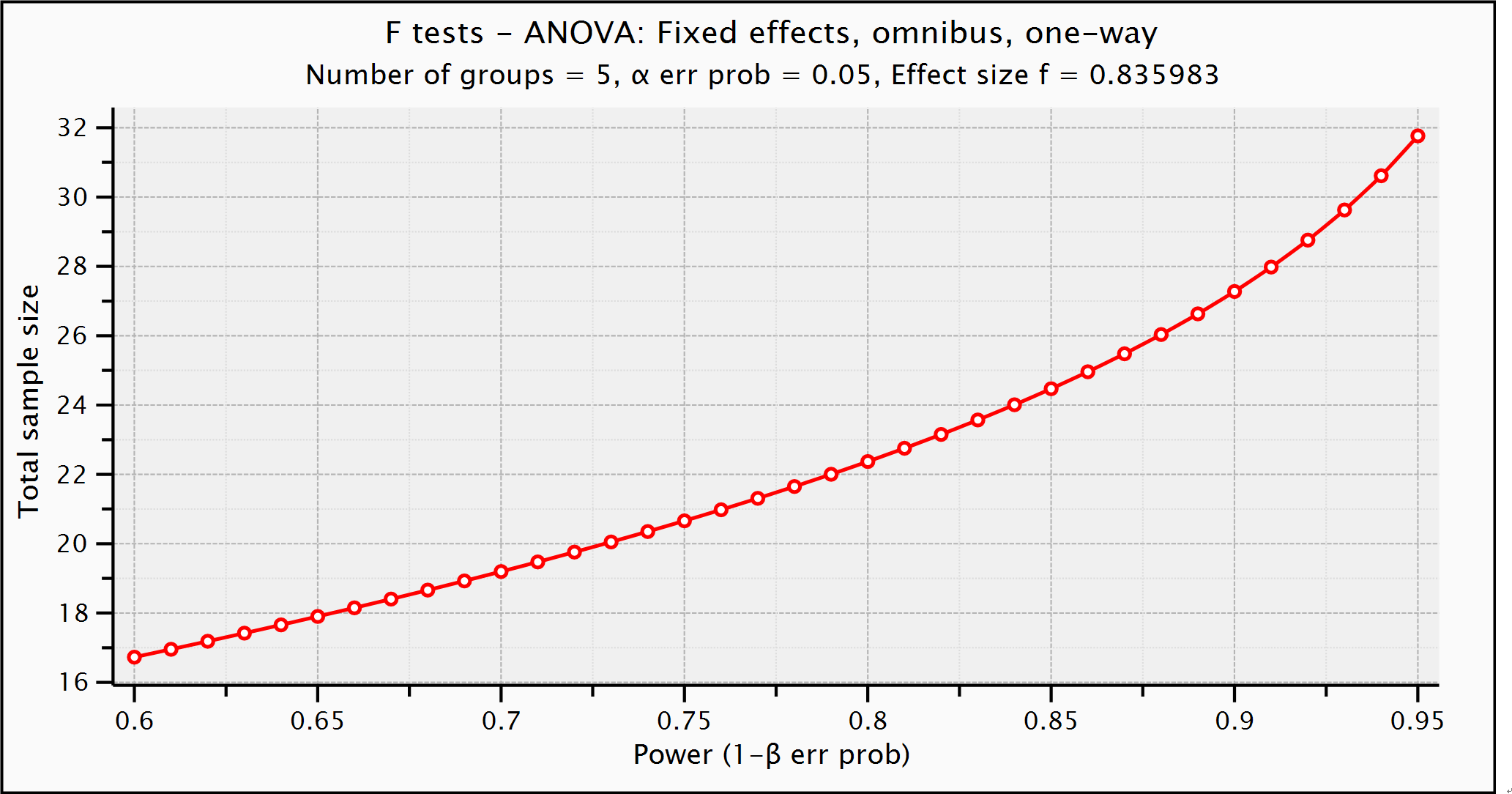


For the requirement of power = 0.8, the total sample size needed would be 25 mice (i.e. 5 mice for each condition). From our current data, i.e. the total mouse amount was 18, it would result in a power = 0.65.

Supplementary Table

**Table S1.** Selected drug optimizations utilized for *in vitro* and *in vivo* examinations.

| Drug combination | *In vitro* dose (μg/ml) | | | *In vitro* cell viability (%) | *CI* | *DRI_ave_*^a^ | *In vivo* dose (mg/kg)^b^ | | |
| --- | --- | --- | --- | --- | --- | --- | --- | --- | --- |
|  | Cis | Pac | Dox |  |  |  | Cis | Pac | Dox |
| ***A*** (nanodroplet platform) | 10 | 0.6 | 20 | 18.2 | 0.2 | 28.4 | 1.1 | 4.2 | 1.2 |
| ***B***  (96-well plate) | 10 | 0.6 | 20 | 20.0 | 0.5 | 9.2 | 4.7 | 17.9 | 5.3 |

^a^The average DRIs (*DRI_ave_*) were evaluated based the equation (3), which results in (45.4+34.4+5.3)/3=28.4 and (5.1+18.6+4.0)/3=9.2 for the combinations *A* and *B*, respectively.

^b^The translation from *in vitro* to *in vivo* doses administered was based on the defined equations (2) and (3). Details can be referred in the Methods.

Supplementary Figures


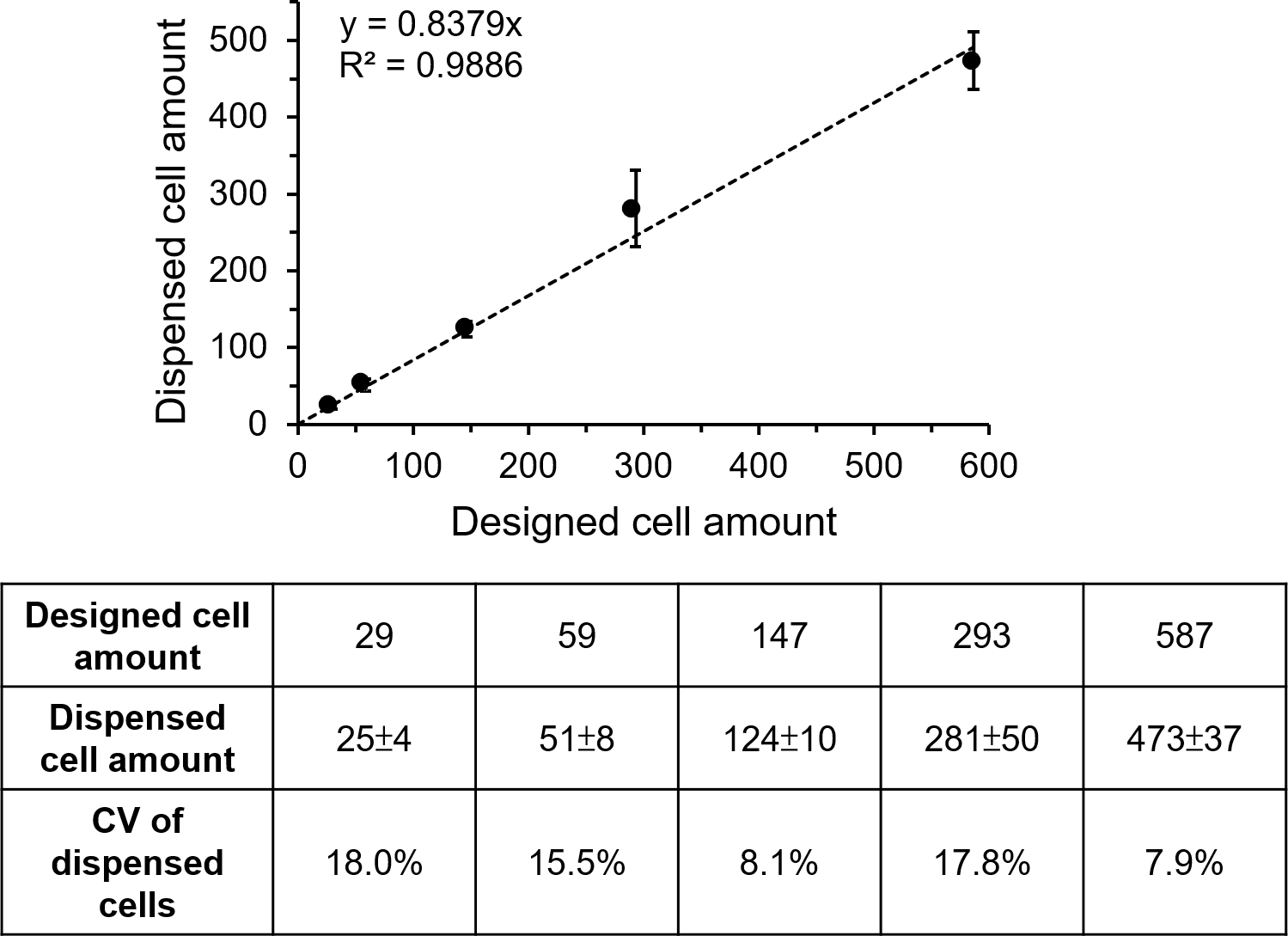


**Figure S1. Uniform cell amount in droplets dispensed from the system.** Top panel shows the linear relationship between the designed and the dispensed cell amount. Bottom panel lists the corresponding cell amount as well as the coefficient of variation (CV) of dispensed cell amount. The average CV from the five conditions is determined to be around 13.4%. Data represents the mean ± SD from 20 dispensed cell droplets. The volume of dispensed droplet is 200 nl.


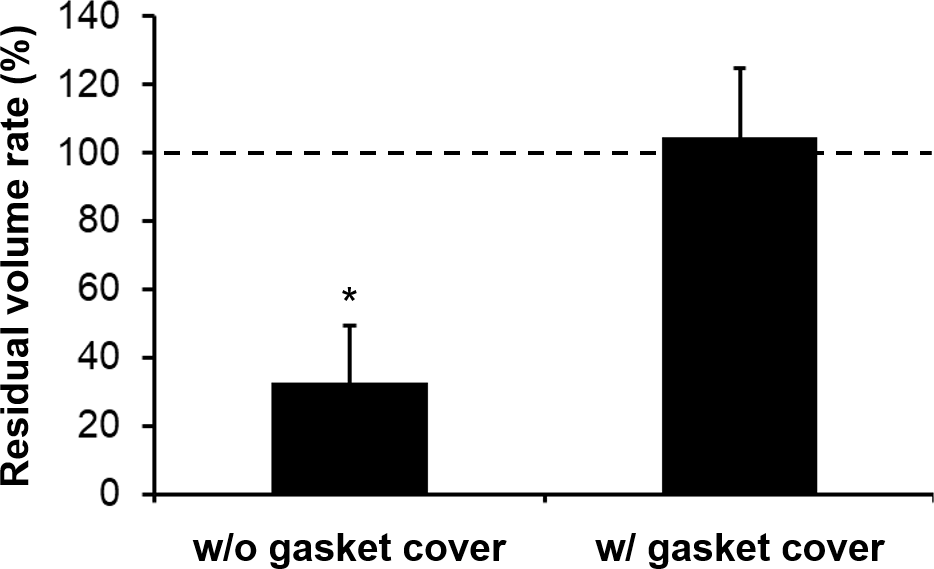


**Figure S2. Comparison of residual volume per droplet dispensed on 9×9 *ParaStamp chip* w/o and w/ PDMS gasket covers after 24 h.** The chips were placed in a humidified 5% CO_2_ incubator at 37°C. The residual volume rate at 24 h was normalized against the initial volume dispensed at 0 h per experiment. Data represents the mean ± SEM from 3 independent experiments (*p < 0.05).


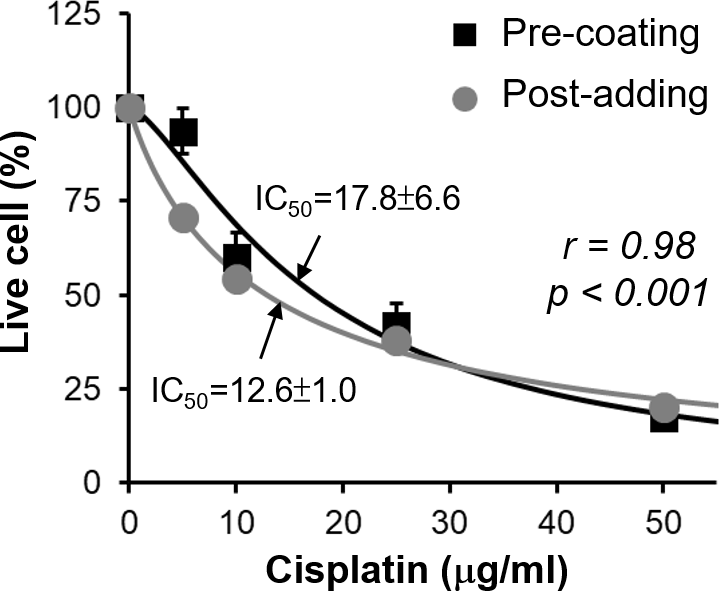


**Figure S3. Comparison of toxicity profiles of 24 h cisplatin treatment on PC-3 cells based on the pre-coating or post-adding approach.** Cells were cultured in 96-well plates and the cell viability was examined by MTT assays. Pre-coating indicates that drugs are pre-coated and then dehydrolyzed onto the corresponding well surfaces prior to cell loading (similar to the approach shown in Figure 3). In contrast, post-adding is loading with drugs following the cell plating in wells, similar to the drug screening approach by 96-well plates described in Methods. There is no significant difference between the two toxicity profiles (Pearson’s r = 0.98, p < 0.001) and their IC_50_ values. Data represents the mean ± SEM from 2 ~ 3 independent experiments.


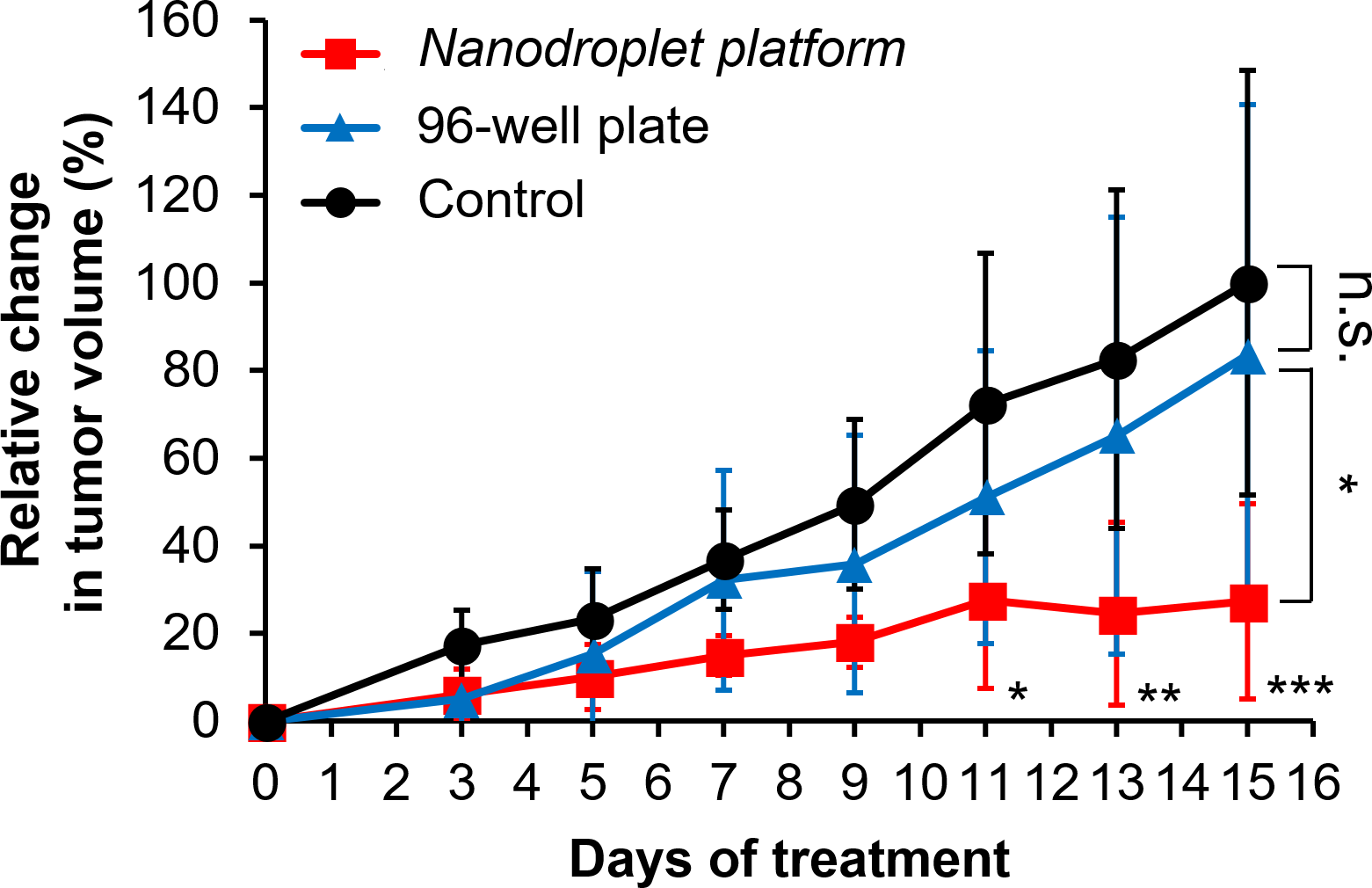


**Figure S4. The selected drug optimization enables to efficiently inhibit PC-3 tumor growth in athymic nude mice.** Comparison of tumor inhibitions by the selected drug optimizations from nanodroplet cell processing platform and the 96-well plate, respectively. The control set was treated with PBS. Details can be referred in Table S1. The change in tumor volume was evaluated as a volume change against the initial volume at day 0. Each data represents the mean ± SD as a percentage of the final volume change of controls at day 15 (n = 4 for both nanodroplet platform and 96-well plate and n = 8 for control set). *p < 0.05, **p < 0.01, and ***p < 0.001 were compared to the controls, except for the indicator.


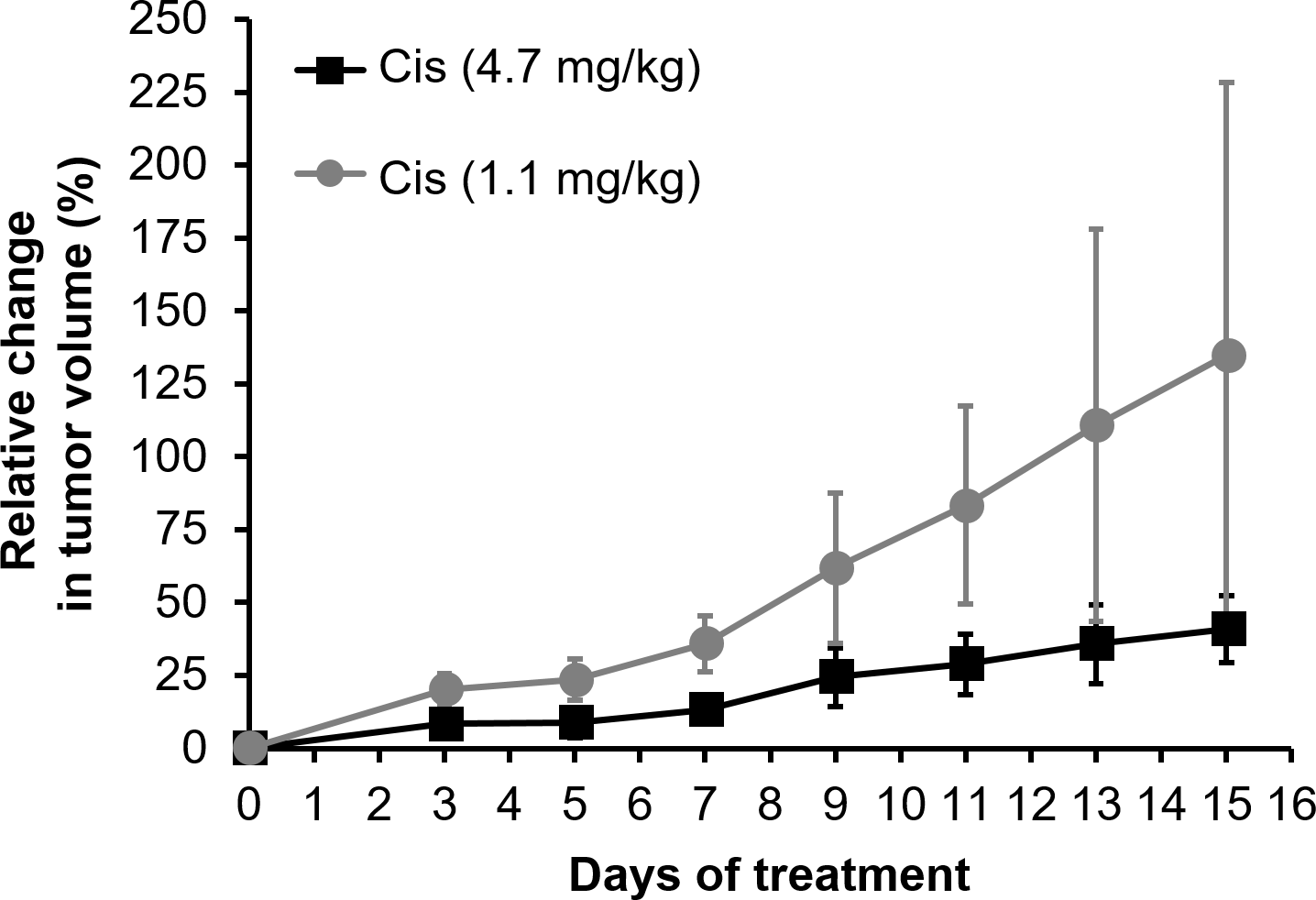


**Figure S5. Inhibition of PC-3 tumor growth in athymic nude mice by the cisplatin administration with two different concentrations.** It indicates that the efficiency of tumor inhibition positively depends on the drug concentration utilized. Each data represents the mean ± SD as a percentage of the final volume change of controls (administered by PBS) at Day 15 (n = 4 for 4.7 mg/kg and n = 2 for 1.1 mg/kg).
